# Monolithic Integration of Nano-Ridge Engineered InGaP/GaAs HBTs on 300 mm Si Substrate

**DOI:** 10.3390/ma14195682

**Published:** 2021-09-29

**Authors:** Yves Mols, Abhitosh Vais, Sachin Yadav, Liesbeth Witters, Komal Vondkar, Reynald Alcotte, Marina Baryshnikova, Guillaume Boccardi, Niamh Waldron, Bertrand Parvais, Nadine Collaert, Robert Langer, Bernardette Kunert

**Affiliations:** 1 Advanced RF Group, IMEC, 3001 Leuven, Belgium; Abhitosh.Vais@imec.be (A.V.); Sachin.Yadav@imec.be (S.Y.); Liesbeth.Witters@imec.be (L.W.); Komal.VondkarKodandarama@imec.be (K.V.); Reynald.Alcotte@imec.be (R.A.); Marina.Baryshnikova@imec.be (M.B.); Guillaume.Boccardi@imec.be (G.B.); Niamh.Waldron@imec.be (N.W.); Bertrand.Parvais@imec.be (B.P.); Nadine.Collaert@imec.be (N.C.); Robert.Langer@imec.be (R.L.); Bernardette.Kunert@imec.be (B.K.); 2 Department of Electronics and Informatics–ETRO, Vrije Universiteit Brussels, 1050 Brussels, Belgium

**Keywords:** nano-ridge engineering (NRE), III–V on Si, MOVPE, hetero-epitaxy, HBT

## Abstract

Nano-ridge engineering (NRE) is a novel method to monolithically integrate III–V devices on a 300 mm Si platform. In this work, NRE is applied to InGaP/GaAs heterojunction bipolar transistors (HBTs), enabling hybrid III-V/CMOS technology for RF applications. The NRE HBT stacks were grown by metal-organic vapor-phase epitaxy on 300 mm Si (001) wafers with a double trench-patterned oxide template, in an industrial deposition chamber. Aspect ratio trapping in the narrow bottom part of a trench results in a threading dislocation density below 10^6^∙cm^−2^ in the device layers in the wide upper part of that trench. NRE is used to create larger area NRs with a flat (001) surface, suitable for HBT device fabrication. Transmission electron microscopy inspection of the HBT stacks revealed restricted twin formation after the InGaP emitter layer contacts the oxide sidewall. Several structures, with varying InGaP growth conditions, were made, to further study this phenomenon. HBT devices—consisting of several nano-ridges in parallel—were processed for DC and RF characterization. A maximum DC gain of 112 was obtained and a cut-off frequency f_t_ of ~17 GHz was achieved. These results show the potential of NRE III–V devices for hybrid III–V/CMOS technology for emerging RF applications.

## 1. Introduction

III–V power amplifiers (PA) are crucial elements in the front-end of transceiver systems for communication technologies. More specifically, III–V heterojunction bipolar transistors (HBTs) and high electron mobility transistors (HEMTs) are known to achieve good performance not only in the low GHz bands, but also over the full mm-wave spectrum, enabling data rates above 10 Gbps [1,2,3,4,5]. Although GaAs/InGaP HBTs grown on expensive GaAs substrates are already being used in PA for mobile phones [6], the heterogeneous integration of III–V compound semiconductors onto a 300 mm Si platform is one of the promising routes to enable hybrid III–V/CMOS technology for RF applications [7,8]. This will reduce cost, power losses, and chip footprint, while, at the same time, improving the flexibility of the circuit design, the overall system performance, and enabling the use of advanced 300 mm CMOS lithography and process capabilities for III–V device fabrication [8,9].

III–V nano-ridge engineering (NRE) is a unique method to monolithically co-integrate low defect density III–V material on Si, without the need for thick, strain-relaxed buffer layers on Si [6,10], or expensive (native) substrates followed by a complex transfer or bonding process [11]. The NRE method begins with aspect ratio trapping (ART) [12] of defects inside a narrow trench, which was already successfully applied in realizing an InGaAs FinFET on 300 mm Si [13]. NRE allows the nano-ridge (NR) to expand its volume outside of the narrow trench with the desired shape, controlled by the growth conditions [14,15], enabling fabrication of large-area devices. An optically pumped distributed feedback NR laser was previously demonstrated by imec [16,17], and, more recently, InGaP/GaAs NR HBTs [18,19], a NR waveguide detector [20], and GaAs NR p-i-n diodes [21] were reported. NRE is also explored for GaSb and InGaAs alloys [22,23]. InGaAs NRE combined with the learning from the InGaP/GaAs NR HBTs is a first step towards enabling InP-based NR HBT technology on a 300 mm Si wafer platform. Specifically, InP HBTs address the challenges related to speed, efficiency, and output power needed for the next generations of wireless high data rate communication systems [24,25,26].

In this work, we report on the growth and structural characterization of the first nano-ridge engineered InGaP/GaAs HBT stack on 300 mm Si wafers with a double trench-patterned oxide template. The observations made on this NR HBT structure prompted the investigation of the InGaP growth conditions, to understand an unexpected and uncontrolled facet change at the top of the NR, after depositing a layer of InGaP inside the oxide template. Therefore, several InGaP test structures, with varying growth conditions and layer thicknesses, were grown and analyzed.

Although the NR HBTs were grown in IMEC’s 300 mm CMOS pilot line, at the moment, devices are processed on wafer pieces in a lab-like environment, for accelerated feedback on device functionality and electrical performance. Gummel plots of two NR HBT structures are shown and compared to a HBT processed on a 2” GaAs substrate. The gain is benchmarked against the literature data of a planar InGaP/GaAs HBT grown on Ge-on-Si. Finally, the RF performance of the fabricated HBTs is also discussed.

## 2. Materials and Methods

**Figure 1:**
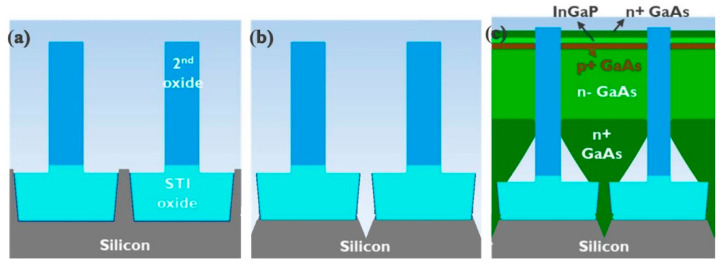
Schematic of the double trench-patterned oxide template (**a**) after opening the wide trench in the second oxide, (**b**) after silicon recess etch to create a {111}-faceted V-groove at the bottom of the narrow trench and (**c**) after epitaxial deposition of a HBT layer stack.

All nano-ridges (NR) shown in this paper were grown by metal-organic vapor-phase epitaxy (MOVPE) using a 300 mm industrial deposition chamber. The following precursors were used: tertiary butylarsine (TBAs), tertiary butylphosphine (TBP) and trimethylarsine (TMAs) for group V elements, triethylgallium (TEGa), trimethylgallium (TMGa) and trimethylindium (TMIn) for group III elements, and carbon tetrabromide (CBr_4_) and silane (SiH_4_) as *p*- and *n*-type dopants, respectively. Growth was conducted on 300 mm Si (001) wafers (n = 1 × 10^19^·cm^−3^) with a double trench-patterned oxide template (Figure 1). This template is based on two separate oxide deposition and patterning processes. Slightly tapered trenches (narrower towards the top), 70 nm by 100 µm along (110), embedded in a first 400-nm-thick SiO_2_ layer with a pitch of 920 nm are fabricated by a shallow trench isolation (STI) process. They are hereafter referred to as narrow trenches. The second oxide layer was deposited by chemical vapor deposition and then polished using chemical mechanical polishing (CMP) to a thickness of 1000 nm. After the trenches about 660 nm wide—hereafter referred to as wide trenches—in this second oxide were opened, centered at the narrow trenches, the Si embedded in the first STI oxide layer could be removed by etching with tetramethylammonium. This led to a {111}-faceted V-shaped bottom of the trench, which prevented the formation of anti-phase domains in the III–V layer. Prior to growth, the native oxide was removed from those {111} Si facets at temperatures below 200 °C in a Siconi™ chamber from Applied Materials. The patterned wafer was then transferred in N_2_ ambient atmosphere into the MOVPE reactor for the III–V deposition step.

The growth of all GaAs-based NRs discussed in this paper start with the following 2 steps at 20 Torr chamber pressure: First, the deposition of a low-temperature seed layer of 20 to 30 nm at 360 °C with a V/III ratio of the group V and III precursors in the gas phase of 60. Due to the low temperature, TEGa was used here to profit from the better decomposition behavior in combination with TBAs. The rest of the layers were grown using TMGa. Secondly, the narrow trench filling with GaAs at 590 °C and a V/III of 30 was conducted. Using nano-ridge engineering, i.e., applying the right growth conditions to obtain the desired NR shape, the growth could then be continued outside of the narrow trench. For a HBT device this means achieving a broad flat (001) NR surface in order to obtain smooth horizontal interfaces between the active layers. The chamber pressure for these layers was 80 Torr.

**Figure 2:**
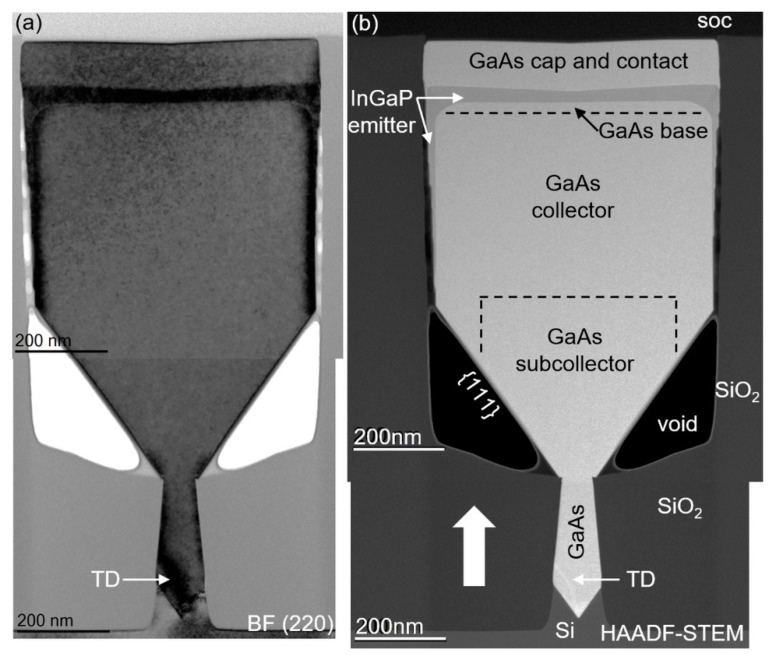
Cross-section (stitched) TEM images of a HBT layer stack viewed under (**a**) two-beam BF (220) and (**b**) HAADF-STEM conditions with all device layers indicated. The broad arrow points to the approximate off-center location for the longitudinal cut shown in Figure 3.

The HBT stack was built up in the following way: first, a widening (up to 430 nm wide) 400 nm high GaAs subcollector (n = 1 × 10^19^ cm^−3^) was grown, followed by a 400-nm-high GaAs collector (n = 5 × 10^16^ cm^−3^), both at 590 °C and with a V/III of 30. The aim was that the NR was touching the second oxide at this point to prevent any further sidewall deposition. Then the temperature was lowered to 550 °C for the growth of the 20 nm GaAs base (*p* = 7.5 × 10^19^ cm^−3^) using TMAs with a V/III of 5. The temperature was then increased again to 580 °C for the deposition of a 50 nm InGaP emitter (V/III = 40, TMIn/III = 0.59 and n = 5 × 10^17^ cm^−3^). The structure was then finished with a 30 nm GaAs cap (n = 5 × 10^18^ cm^−3^) followed by a 50 nm GaAs contact (n = 1 × 10^19^ cm^−3^), both deposited with a V/III of 30. These layers are represented schematically in Figure 1c and are more realistically indicated in the HAADF-STEM image of Figure 2. The growth conditions of the InGaP test structures are mentioned in the Results and Discussion section.

Structural characterization was carried out by scanning electron microscopy (SEM) and transmission electron microscopy (TEM). Cross-sectional TEM specimens were prepared using a focused ion beam (FIB) in a cut direction perpendicular and along the NR orientation. The samples were capped using an amorphous spin-on carbon (soc) layer and a thin Pt layer was sputtered as an additional protection layer. The TEM inspection was conducted with a double-corrected Titan3 G2 60–300 system from FEI at voltages of 120 to 200 kV. To visualize all possible crystal defects very different imaging conditions were applied, such as high-resolution TEM, two-beam bright-field (BF) TEM, high-angle annular dark-field scanning transmission electron microscopy (HAADF-STEM, contrast is proportional to lamella thickness and to the square of the average atomic number of the layer), dark-field STEM (DF-STEM, contrast is related to the crystallinity and density of the layers) along with energy-dispersive X-ray spectroscopy (EDS), to explore the atomic alloy distribution. The degree of relaxation of the NRs and composition of InGaP were extracted from high-resolution X-ray diffraction reciprocal space maps (RSMs) around the (224) reflection, recorded with the incident beam parallel and perpendicular to the trench orientation.

## 3. Results and Discussion

### 3.1. Structural Analysis of InGaP/GaAs HBT Stack

The key advantage of NRE is the separation of the active device region in the wide trench, from the area of strain relaxation and defect formation, due to the lattice mismatch between GaAs and Si inside the narrow trench. Hence, an in-depth structural characterization of the complete HBT stack is an important aspect to confirm the advantage of our novel integration approach. The HBT stack, as described in the Materials and Methods section, is analyzed by TEM, applying different viewing conditions to detect all possible crystal defects. Two TEM images, taken across the NR, are shown in Figure 2. All device layers are indicated in the HAADF-STEM image, where a clear contrast difference between GaAs and InGaP is visible. It is noticed that the GaAs NR was not touching the oxide when the deposition of the InGaP emitter was started, which results in some InGaP growth on the sidewalls. Upon close inspection, InGaP step bunching is observed on the {110} sidewalls. We believe that as the gap between the NR and the oxide reduces to a certain value, the group III and group V material transport via gas-phase diffusion ceases to contribute to the growth, and only the surface diffusion component is left. The BF (220) image is more suitable to reveal strain fields and crystal defects. At the bottom of the trench, the lattice mismatch between GaAs and Si is accommodated on the {111} facets, by formation of a 60° misfit dislocation array at the interface, as we reported previously [27,28]. The strain field of a threading dislocation (TD) is clearly visible in that region. One TD is also noticeable in the HAADF-STEM image, which is trapped by aspect ratio trapping, resulting in TD-free material at the top of the narrow trench. Inspecting four adjacent NRs, no indication of TDs is found in the NR volume inside the wide trench, and no dislocations are generated at the InGaP/GaAs interfaces.

**Figure 3:**
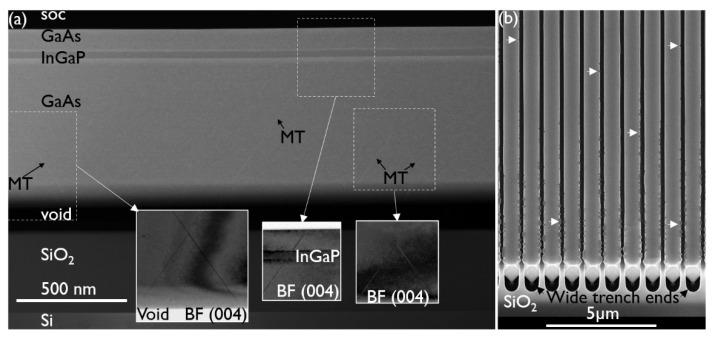
(**a**) DF-STEM image of an off-center cut along a trench. The contrast change above the void is due to the increasing material thickness at the (111) facet. Some micro-twins (MTs) are faintly visible. The BF (004) insets (on the same scale) show a part of the MTs more clearly. (**b**) Tilted top-view SEM image of NRs at the end of the wide trench pattern where the NRs have a {111} facet. The arrows indicate example locations of defectiveness on the NR sides.

The TEM specimen along the length of the NR was cut off-centered at the position of the broad white arrow in Figure 2b. In Figure 3a, the same observation can be made as in Figure 2. No TDs are observed in the device layers and no TDs are generated at the InGaP/GaAs interfaces. However, some micro-twins (MT) running through the whole stack are faintly visible in the DF-STEM image. The inset images taken under two-beam BF (004) conditions (on the same scale) show them more clearly. The observed MTs originate in the seed and cannot be trapped by aspect ratio trapping, as they are planar defects (PD) that run along the trench. Improving the seed growth conditions should allow us to further reduce the PD density. In addition, we have observed that the PD formation is also strongly correlated with the trench formation and pre-epi cleaning processes. Hence, both the seed deposition parameters as well as the template fabrication must be further optimized to reduce the MT density to below 0.2 μm^−1^–0.5 μm^−1^ [22]. It is important to note that a PD reaching the side and top surfaces of the NR is not accompanied by partial dislocations and, hence, should not significantly impact the device performance.

**Figure 4:**
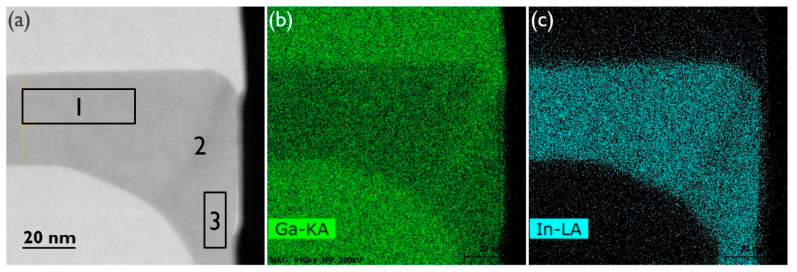
(**a**) HAADF-STEM image of an InGaP corner between 2 GaAs layers, and EDS maps of that corner based on (**b**) Ga-Kα and (**c**) In-Lα X-ray emission. The In composition of regions 1, 2 (the darker vein) and 3 is deduced as the following: 42%, 33% and 40%, respectively.

The InGaP/GaAs interfaces and top surface are flat. The contrast change at the bottom of the GaAs layer, just above the void, is related to the change in material thickness at the (111) facet, i.e., thinner towards the void. The top surface appears flat in the 5 μm sample that was inspected by TEM. However, the top-view SEM image in Figure 3b shows that there is some defectivity at the NR sides, which is more pronounced closer to the end of the trenches. At the trench ends, there is additional material loading from the oxide pattern that contributes to the locally enhanced growth rate of the NR in these regions. The enhanced growth rate causes the InGaP layer to come into contact with the oxide sidewall sooner, which, as will be discussed in a later section, results in defect generation. As the features are not large and sparsely distributed along the NR sides, they were missed in the TEM samples shown in Figure 2 and Figure 3. Asymmetrical (224) reciprocal space maps (RSMs) showed a weak signal from the thin InGaP layer, and an In mole fraction of about 0.41 could be deduced. The InGaP composition was also determined by EDS. For the top facet, region 1 in the HAADF-STEM image of Figure 4, an In mole fraction of 0.42 was estimated, which matches well with the composition extracted from the (224) RSMs. The following two interesting observations were made: First, region 2 in Figure 4a marks a darker vein in the InGaP layer running from corner to corner. The indium percentage here is only 33%, hence, the vein is Ga-rich. The vein extends from the corner as the NR grows. This compositional difference can also be observed in the EDS maps of the Ga-Kα (Figure 4b) and In-Lα (Figure 4c) X-ray emissions. Such a vein, or trace of the NR corner evolution during growth, was also observed for InGaAs NRs [23] and is caused by a dependency of the group III incorporation on the NR facet. Secondly, region 3, i.e., the (110) sidewall, has a slightly lower In mole fraction of 0.40, compared to the (001) top facet. This may also point to a different incorporation efficiency for Ga and In atoms on the (110) facet, or to a less efficient supply of the In species to the sidewall.

### 3.2. Systematic Growth Study into the Origin of the Observed Defectiveness

As the defectiveness of the NR sides observed in the top-view SEM image of Figure 3 does not yet extend far towards the NR center, it must be formed relatively high in the structure. Although the TEM samples did not show TD generation at the InGaP/GaAs interfaces, a 5 × GaAs/InGaP multilayer stack was deposited at 590 °C, to test diverse growth conditions. The different GaAs layers were all grown with a V/III of 30 and a fixed Ga-flux, but varying n-doping, to explore the possible impact of the presence of silane and/or the Si-doping level. All the InGaP layers had a TMIn/(TMIn + TMGa) of 0.57 and V/III of 40, except for the fourth layer, where the V/III was 80. In the fifth layer, the double growth rate was used. N-doping was an additional (random) parameter in the InGaP layers. Table 1 presents the conditions.

**Figure 5:**
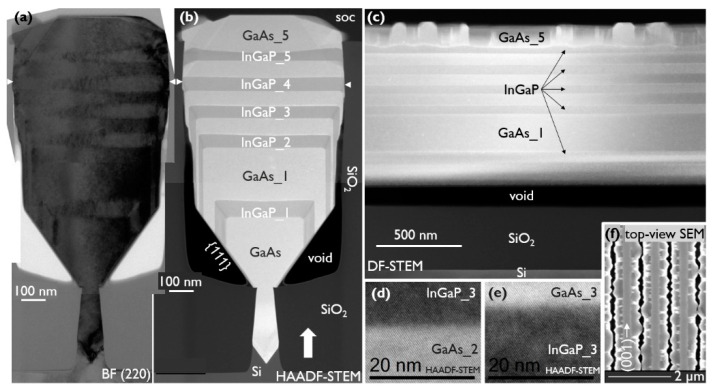
TEM of a 5 × GaAs/InGaP multilayer stack cut across the trench under (**a**) two-beam BF (220) and (**b**,**d**,**e**) HAADF-STEM and (**c**) of an off-center cut (at broad white arrow in (**b**)) along the trench under DF-STEM. The full NR images (**a**,**b**) are stitched. High-resolution images of the third InGaP layer’s interfaces are shown in (**d**,**e**). (**f**) Top-view SEM of NRs with defectivity at the NR sides and the pristine (001) top facet present in between.

Figure 5a,b show a cross-section TEM image of the multilayer stack under two-beam BF (220) and HAADF-STEM, respectively. The contrast in the HAADF-STEM image allows us to clearly identify the individual layers and match them with the BF image. Starting at the bottom of the narrow trench, the 60° misfit dislocation array can be recognized upon closer inspection of the {111} facets in the BF image. TDs are then filtered out by ART, and the GaAs is finally TD-free, once grown outside of the narrow trench. We do not observe any evidence of TDs penetrating the multilayer stack, nor are new TDs being generated at the GaAs/InGaP interfaces. These interfaces are sharp and defect-free, as exampled by the high-resolution STEM images of the third InGaP layer in Figure 5d,e. The InGaP layers are well lattice matched to GaAs, based on a (224) RSM, with an average In mole fraction of 0.48, deduced from the EDS analysis. The interfaces between the different layers are found to be flat up to the fourth InGaP layer. This is the first layer that contacts the oxide, and we notice the formation of planar defects along the {111} planes, as indicated by the small arrows in both the BF and the HAADF-STEM images. These planar defects continue through the rest of the stack, all the way to the top of the structure. The thicker the deposition after their formation, the wider the sideways extent of the defects and, hence, the narrower the remaining portion of the original (001) facet in the center of the NR, until this facet eventually disappears. It is observed that GaAs_4, InGaP_5, and GaAs_5 no longer have a flat top facet, but clearly different surface planes are present at the NR sides. This is the result of twinning, which will be shown in more detail later. Figure 5c is a DF-STEM image of a section from a 4-µm-long sample along the NR, but off-center at roughly the position of the broad white arrow in Figure 5b. In this direction, there is also no evidence of TDs being present in the layers grown in the wide trench, and the interfaces are flat and horizontal. However, similarly to the HBT stack, some MTs were found in the longitudinal direction. The individual InGaP layers are indicated in the image. Again, the vertical contrast change just above the void is linked to the presence of the {111} facet. The bottom interface of InGaP_5 is still flat, because the defective area at the NR side did not extend to this interface in the TEM specimen yet, whereas the top interface of InGaP_5 does show distinct roughness. Finally, the top GaAs layer is twinned in all directions.

Looking at the HAADF-STEM image (Figure 5b), one easily notices that the first three InGaP layers grow in a nice box shape around each GaAs layer. For InGaP_3, the sidewall deposition reduces in thickness towards the bottom of the NR. For GaAs_4, step-bunching can be clearly observed on the sidewall. As InGaP_1 and 2 were undoped and InGaP_3 is doped more than the emitter in the HBT stack for the same growth conditions, we conclude that the emitter doping concentration was not the cause for the sparse defectiveness observed on the HBT stack, but was rather due to the InGaP emitter locally contacting the oxide, which then induced the twinning. The presence of Ga-rich veins in the first three InGaP layers is unmistakable. On some locations in the BF image, the signatures of their induced strain fields can also be recognized.

Finally, Figure 5f is a top-view SEM image of several NRs, revealing recurrent surface modulation. In the middle of each NR, a band of uniform contrast reveals the presence of the pristine (001) top facet of the NRs. The twinned material is found on either side. As mentioned before, the width of the pristine (001) top facet depends on the deposited layer thickness after InGaP contacts the oxide.

**Figure 6:**
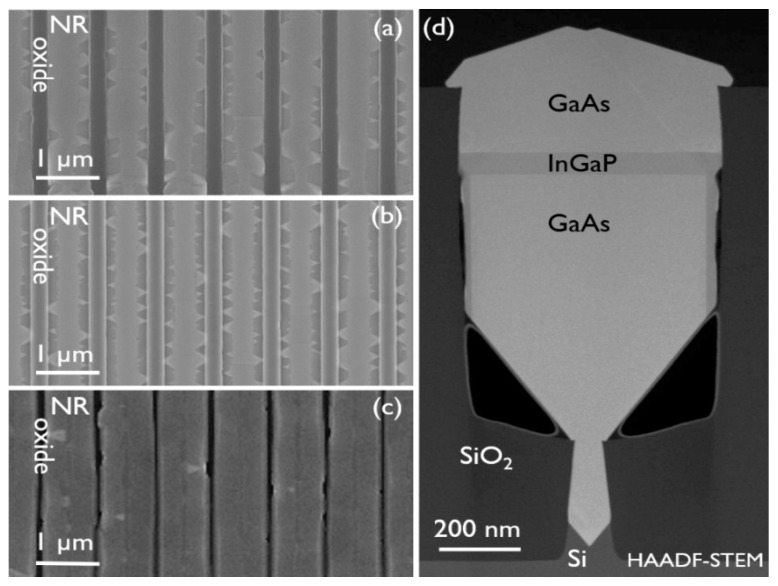
Top-view SEM images of NRs with a single thin InGaP layer (**a**) grown at 590 °C with double growth rate, (**b**) grown at 590 °C with In mole fraction of 0.68 and (**c**) grown at 500 °C. (**d**) Cross-section HAADF-STEM image of the sample shown in (**c**).

Many different InGaP growth conditions, such as growth rate, deposition temperature, reactor pressure, V/III ratio, as well as the In mole fraction in the solid, were varied in additional deposition experiments of GaAs NRs with a single thin InGaP layer in contact with the oxide, but none of them prevented the twinning. Some representative examples are shown in Figure 6. Twinning at the NR sides is clearly observed by top-view SEM in Figure 6a (double growth rate) and Figure 6b (higher In mole fraction of 0.68). In the top-view SEM image of a sample with InGaP grown at 500 °C (Figure 6c), it is difficult to recognize the twinning. At first glance, the NR appears to have (111) facets, but the cross-section HAADF-STEM image of that NR (Figure 6d) reveals multiple facets at the top. The GaAs layer grown on top of InGaP was thick enough to eliminate the (001) facet from the top surface.

As changing the InGaP MOVPE growth conditions over a large parameter range did not prevent the planar defect formation, we investigated more specific GaAs/InGaP/GaAs NR heterostructures, to study the twinning phenomenon in more detail.

**Figure 7:**
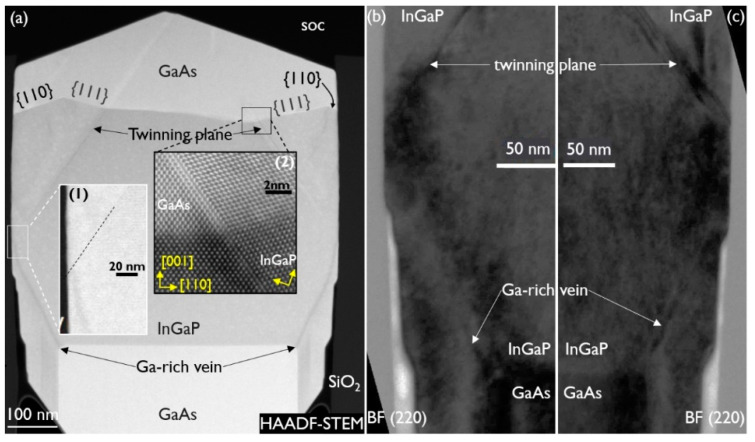
(**a**) Cross-section HAADF-STEM image of a GaAs/InGaP/GaAs NR grown at 590 °C. Inset (1): magnification of site of origin of the twinning plane (indicated by the dashed line). Inset (2): high-resolution image of the GaAs/InGaP interface across the twinning plane. Magnification in BF (220) conditions of left (**b**) and right (**c**) side of the NR shown in Figure (**a**) where the twinning plane originates in the InGaP layer at the oxide wall.

In an NR heterostructure, using the “InGaP_2” growth conditions from the multilayer sample, the InGaP layer thickness was significantly increased, to better observe what happens in proximity of the oxide. Figure 7a shows the NR in HAADF-STEM conditions. The GaAs and InGaP regions with an In mole fraction of 0.50 are easily recognized, based on the contrast difference. The Ga-rich veins follow the NR corners as the NR widens during growth. When the veins seem to touch the oxide, planar defects along the {111} planes are formed. However, as shown in the magnified image of that region (see inset (1)), the planar defect seems to be initiated when the Ga-rich vein is not yet touching the oxide. Due to the pronounced composition change inside the Ga-rich vein, we hypothesize that the induced strain fields, reaching out to the oxide surface, already initiate the twinning, although the vein does not touch the oxide yet.

Inset (2) shows a high-resolution image of the GaAs/InGaP interface across the planar defect. The undisturbed and twinned lattice are resolved in detail, revealing a clear crystal rotation. As a result, the top facets of the NR are not part of the original crystal anymore, which has almost disappeared at the top. They are, in fact, the (110) facets of the twinned crystal. As there are also (111) planes present at the GaAs/InGaP interface, this points to a slow growth rate for (110) compared to (111) in GaAs. Figure 7b,c are BF (220) images of the left and right NR sidewall, respectively. The Ga-rich vein and the beginning of the twinned region are indicated in both figures. No other misfit defect is observed where the Ga-rich vein is in proximity of the oxide and the twin originates.

**Figure 8:**
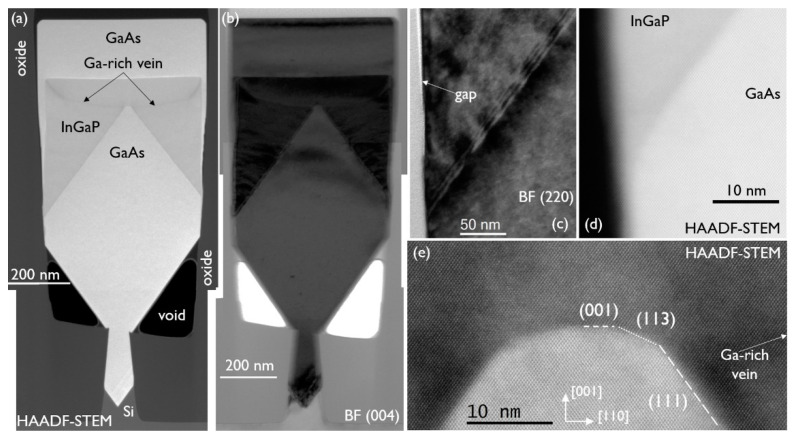
(**a**) HAADF-STEM and (**b**) BF (004) cross-section TEM image of a GaAs/InGaP/GaAs NR with a {111}-faceted starting GaAs NR. Zoom-in on the InGaP/GaAs interface at the oxide under (**c**) BF (220) and (**d**) HAADF-STEM conditions. (**e**) Zoom-in on the InGaP/GaAs interface at the tip of the {111}-faceted GaAs NR.

To confirm a correlation between the presence of the Ga-rich vein and its strain fields and twin formation, a thick InGaP structure was grown where the starting GaAs NR was {111} faceted. The growth conditions for the undoped InGaP layer were the same as for the above discussed structure with a thick InGaP layer (grown on box-shaped GaAs), and they are as follows: 590 °C, V/III of 40, and TMIn/(TMIn + TMGa) of 0.57. The stack was then capped with a GaAs layer, grown at 590 °C, with a V/III ratio of 30. Cross-section HAADF-STEM and BF (004) images of the stack are shown in Figure 8a,b, respectively. The {111}-faceted GaAs NR is recognized in both images. After the InGaP deposition, the NR evolved into a box shape. Although the InGaP initially contacts the oxide, it “pulls away” during further growth of the layer, as GaAs from the cap layer deposition can be observed between the oxide and InGaP, towards the top of the InGaP box. Apart from the dislocations at the bottom of the narrow trench, the BF (004) image does not show any dislocations in the bulk of the (wide) NR. Twinning planes are also not observed in this stack. The BF (220) zoom-in (Figure 8c) of the {111} plane at the InGaP/GaAs interface at the oxide wall proves that there is contact between InGaP and the oxide before a gap is formed, but no evidence of twinning is observed. The fringes that are observed parallel to the interface are due to the tilt of the sample required for this two-beam inspection, and the resulting overlap between InGaP and GaAs in the thickness of the sample close to the interface. The HAADF-STEM image in Figure 8d shows a defect-free InGaP/GaAs interface and sidewall.

The trajectory of the Ga-rich vein is clearly visible in Figure 8a. The growth rate on the {111} facets is so high that first the empty volume is almost filled up, widening to form a quasi-box shape, after which the normal box growth takes over, with growth in the (001) direction and slower growth on the {110} facets. At that point, the Ga-rich vein also starts to follow a more expected, steeper path, as observed in Figure 7a. A (224) RSM showed the composition close to being lattice matched to GaAs and from EDS analysis above the Ga-rich veins, an In mole fraction of 0.46 was deduced. EDS analysis below the Ga-rich veins showed that the composition was the same. Figure 8e is an HAADF-STEM image of the GaAs tip. It looks like the tip has short segments of {113} and (001). Inspection of the image by inverse fast Fourier transform did not reveal any misfit defect at the interface. The Ga-rich vein is formed at the top of the {111} facet, at the corner with the {113} facet, and then moves outward, with the deposition on {111} filling up the empty volume. Reviewing Figure 4 again, one can observe the same mechanism, as follows: the Ga-rich vein develops at the corner of the {113} and {111} facets. As the sample of Figure 8 is free of any twin generation, although InGaP is in contact with the oxide, our hypothesis that the presence of the Ga-rich vein close to the oxide induces the planar defect via its strain field is supported.

**Figure 9:**
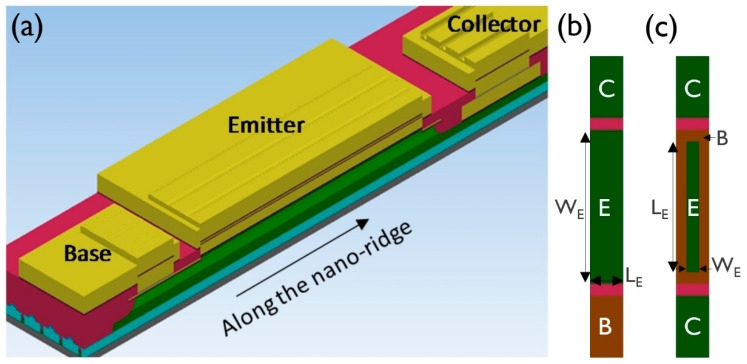
(**a**) Tilted top-view schematic of a multi-NR HBT showing emitter (E), base (B) and collector (C) contacts and a cross-section view of one NR; (**b**) top-view layout of an individual NR without the contact metals present showing the definition of emitter width W_E_ and length L_E_; (**c**) definition of W_E_ and L_E_ in the case of a 300 mm sub-250 nm device process flow.

Since these twins are only present in the emitter (InGaP) and contact (GaAs) layer of our HBT NR devices, their density is very low, far from the trench ends (see Figure 3b), and the lateral extension of the twin at the contact/emitter interface is limited to 35 nm (reaching a maximum of 90 nm at the contact layer top surface) on each side of the NR; therefore, we do not expect a significant impact on the HBT device performance. One way to prevent the twinning, if no suitable growth conditions can be found, would be to start the emitter deposition outside of the second oxide, so the InGaP does not touch the sidewall. Another reason why we are not concerned about this phenomenon is that, in a full 300 mm device process flow, the structure would be etched down to the base at the NR sides, leaving only a 200–300 nm broad section of emitter and contact in the middle of the NR (see Figure 9c in the next section), which is still untwinned.

### 3.3. InGaP/GaAs HBT Device Performance

In the following section, the DC characteristics of two NRE HBT stacks on Si and those of our planar device, processed on a 2” GaAs substrate (planGA), are presented. The current gain will be compared to the literature data of a planar InGaP/GaAs HBT, processed on Ge-on-Si (lit-Ge/Si). This device was grown on a ~1-µm-thick Ge-on-Si buffer, with a threading dislocation density of 2 × 10^7^ cm^−2^ [29]. Our device stack on 2” GaAs was grown with nominal values of doping levels and layer thickness, similarly to the reference NR HBT described and analyzed in the previous section, except that the base doping level was 1 × 10^20^ cm^−3^. The second NR HBT stack (NRbase, base: *p* = 3 × 10^19^ cm^−3^) and the reference one (NRref, base: *p* = 7.5 × 10^19^ cm^−3^) only differ in base doping level. The NRref device consists of 22 NRs in parallel, and the NRbase device consists of 9 NRs, both with an emitter width W_E_ of 35 µm and an emitter length L_E_ of 0.66 µm (width of the wide trench) per NR, as illustrated in Figure 9a,b. The planGA device has an area of 75 × 75 µm^2^. The contact resistivity values extracted from TLM measurements on our samples are 1.2, 1.0, and 0.6 × 10^−5^ Ω∙cm^2^ for *n*-GaAs, and 0.8, 3.6, and 0.6 × 10^−6^ Ω∙cm^2^ for *p*-GaAs material, for NRref, NRbase, and planGA, respectively. The structures were shown to have some PDs originating in the emitter, and running through the emitter cap and contact layer to the top surface, as observed for the HBT structure discussed in Figure 3. These PDs consist of multiple micro-twins and stacking faults with restricted extent, thus partial dislocations are present. Although these partial dislocations do not intersect any critical device junction, they might have a small impact on the performance of the devices processed on wafer pieces in the lab. For scaled devices (W_E_ < 250 nm), integrated in a full 300 mm process flow, the PDs would not be an issue. In these scaled devices, the emitter mesa would be located within an NR with a base all-around device architecture. The emitter layers would be etched down to the base, leaving an emitter with W_E_ of sub-250 nm in the middle of the NR (see Figure 9c). As the PDs only extend 90 nm from either side at the NR top surface, the twinned regions would be completely removed in this device process flow.

**Figure 10:**
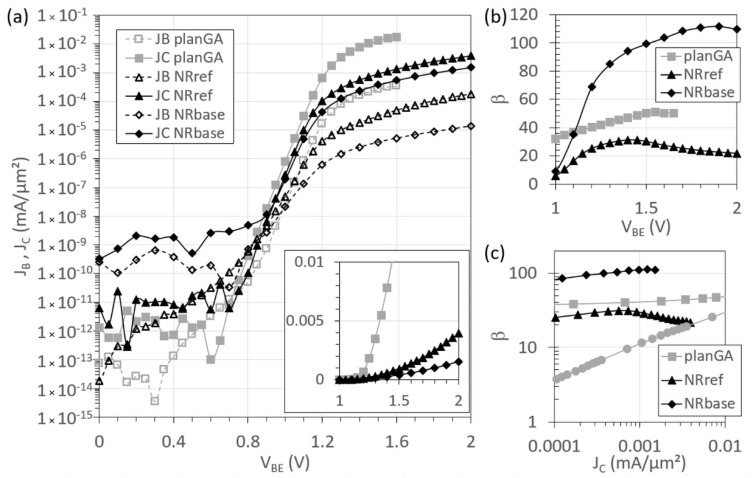
DC electrical characterization of our planar HBT on GaAs (planGA), the reference NR HBT on Si (NRref) and the NR HBT with lower *p*-doped base on Si (NRbase). (**a**) J_B_, J_C_ (inset: J_C_ on linear scale) and (**b**) current gain β as function of V_BE_, measured at V_CB_ = 0. (**c**) Benchmarking DC current gain with respect to collector current density to literature data of an InGaP/GaAs HBT on Ge-on-Si (lit-Ge/Si) [29].

Figure 10 presents the Gummel plots with the base-collector junction at zero bias (V_CB_ = 0 V), and the DC gain of these devices. The ideality factors of the collector current density, J_C_, versus the base-emitter voltage extracted from Figure 10a, are close to one (1.0, 1.1, 1.3) for the three devices. While the ideality factor of the base current density, J_B_, versus the base-emitter voltage for planGA is still close to one, those for the NR devices are much larger, as follows: 1.6 for NRref and 2.0 for NRbase. This is due to a large recombination current at the NR sidewall in the base-emitter space-charge region [19]. The ON current of the NR devices is lower than that of the planar device, due to series resistance effects, as observed in the inset of Figure 10a, where J_C_ is plotted on a linear scale. The NRref base current is higher than that of NRbase, due to the different base doping levels. The leakage current of NRbase is higher, due to a fabrication issue. When the collector is biased by using the chuck instead of the top collector contact, a comparable leakage current is obtained. Figure 10b shows the current gain as a function of V_BE_. The maximum gain for these devices is 50, 31, and 112 for planGA, NRref, and NRbase, respectively. For NRbase, lower base doping was used, to enable a higher current gain. In the NRref curve, the current crowding manifests itself for V_BE_ > 1.6 V, reducing the gain, and in the case of NRbase for V_BE_ > 1.9 V. Although the ON current, at a certain V_BE_, is lower for NR devices than for the planar device, a higher gain could be achieved with the NR devices. In Figure 10c, we compare our devices to the lit-Ge/Si HBT, and it can be observed that NRE results in a better-performing device than growing on a Ge-on-Si buffer, due to the much lower defect density in the active III–V device layers.

Besides the lower defect density and the thinner III–V buffer needed to grow NR HBT layer stacks on Si by NRE—compared to a planar strain-relaxed buffer approach—the nano-ridge design also facilitates the integration of passive devices in high-resistivity (HR) Si. The selective area growth technique inhibits the in-diffusion of group V and/or group III atoms in the regions of the HR Si surface protected by oxide, thus avoiding resistivity reduction, due to unintentional doping. This will prevent degraded performance of the passive devices under the oxide mask. More advantages of NRE in an oxide trench pattern are as follows: the NRs are naturally isolated mesa-structures, which makes them very suitable for multi-finger HBT devices; the wafer bow is kept low, which is important for the subsequent lithography steps and processing, and cracks in the III–V material have not been observed. The width of the NR, however, could be a potential disadvantage for making a collector contact on the side of the HBT device (along the L_E_ direction, see design layout in Figure 9c), instead of at the edges. Being able to access the NR sidewall, to apply the right sidewall surface passivation, might also be key for the device performance.

Table 2 presents a comparison of our NR HBTs to other devices that are reported in the literature as having a GaAs or AlGaAs base. Because the base doping levels and thicknesses are not the same, or a base-collector bias voltage was used, a fair comparison of these devices is challenging. The data summarized in the table show that a low TDD is necessary to reach a high DC gain. The gain can be further boosted by applying either a doping gradient or a composition gradient (e.g., AlGaAs [30,31]) in the base, creating a quasi-electric field to reduce the base transit time.

**Figure 11:**
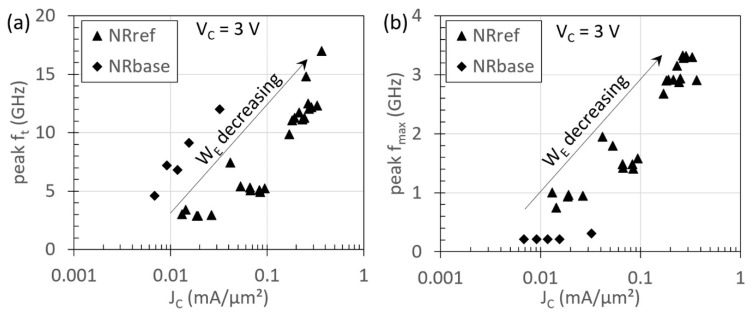
RF small-signal performance of nano-ridge engineered HBTs, consisting of different number of NRs. (**a**) Peak f_t_ and (**b**) f_max_ as a function of current density for devices from NRbase and NRref samples. The data shown are for W_E_ in the range of 8 to 30 µm for NRref (A_E_ 35 to 1334 µm^2^) and 15 to 35 µm for NRbase (A_E_ 160 to 627 µm^2^). Peak f_t_ exceeding 15 GHz are achievable for devices with W_E_ ~8 µm. f_max_ for NRbase is quite small as compared to NRref devices, owing to larger base doping in the latter.

The cut-off frequencies, f_t_ and f_max_, for several HBTs with varying W_E_ were extracted from S-parameter measurements. In Figure 11a, the peak f_t_ for the NRref and NRbase devices is shown as a function of J_C_. Due to the emitter crowding effect, J_C_ is larger for HBTs with smaller W_E_, which, in turn, leads to improved f_t_. At W_E_ ~8 µm, an f_t_ of ~17 GHz is achieved, which is comparable to the f_t_ reported for GaAs-on-Si HBTs in the literature [34]. It is noted that f_max_ (Figure 11b) for the devices investigated here are significantly lower than the f_t_. This is attributed to the large W_E_ and base contact size in these devices, which leads to large base resistance and base-collector capacitance. In addition, the f_max_ for NRbase HBTs is much lower than for NRref HBTs, due to a lower base doping in NRbase HBTs, which results in larger base sheet resistance. In this dataset, devices existing with a different number of NRs were used, but a correlation between the number of NRs and performance has not yet been found.

These values demonstrate that the NRE approach holds great promise, although the RF performance of these devices is still limited, and requires process optimization as well as device scaling. Especially, the scaling of W_E_ to sub-250 nm dimensions, in a 300 mm process flow (see Figure 9c), will help to further reduce the emitter crowding effect and base resistance, leading to improved RF performance.

## 4. Conclusions

Using NRE, InGaP/GaAs HBTs were, for the first time, successfully integrated on a 300 mm silicon platform via selective area growth in an industrial MOVPE reactor. The device stack showed restricted twinning at the top edges of the NR after the InGaP emitter contacted the silicon oxide template. A study of the InGaP growth conditions was unsuccessful in finding a solution to the defect formation phenomenon. It is hypothesized that the strain field from the Ga-rich vein, which is formed in the ternary InGaP alloy, plays an important role in the generation of the twins when it is in proximity of the oxide sidewall. InGaP/GaAs HBTs with two different base doping levels were fabricated on Si. Due to the low TDD, NR HBT devices with a DC gain as high as 112 are possible by reducing the base doping. Devices with shorter W_E_ have higher f_t_, reaching ~17 GHz at 8 µm, due to a lower emitter crowding effect. f_max_ was limited to below 5 GHz. For both devices, f_t_ and f_max_ can be improved by further scaling W_E_.

The material characterization outcome and device results discussed here demonstrate the enormous potential of nano-ridge engineering-based HBTs in front-end modules, for applications beyond 5G. In combination with the learning from InGaAs NRE [23], our work presented here is a promising first step towards enabling InP-based NR HBT technology on a 300 mm Si wafer platform.

## Figures and Tables

**Table 1:** Growth parameters of the layers in the 5 × GaAs/InGaP multilayer stack with TMIn/(TMIn + TMGa) = 0.57 for InGaP. Whether the layer is in contact with the sidewall oxide or not is also specified.

**Layer**	**V/III**	**N (cm^−3^)**	**Remark**
GaAs	30	8 × 10^18^	no contact
InGaP_1	40	undoped	no contact
GaAs_1	30	2 × 10^16^	no contact
InGaP_2	40	undoped	no contact
GaAs_2	30	2 × 10^16^	no contact
InGaP_3	40	9 × 10^18^	no contact
GaAs_3	30	2 × 10^18^	no contact
InGaP_4	80	undoped	contact
GaAs_4	30	undoped	contact
InGaP_5	40	undoped	contact, 2 × growth rate
GaAs_5	30	undoped	contact

**Table 2:** Overview of HBTs with GaAs or compositionally graded (Al mole fraction increases from 0 to 0.10 or 0.12) AlGaAs base listing type of substrate, threading dislocation density (TDD), emitter area (A_E_) and material, base doping and thickness, maximum DC current gain (β_max_), the collector current density (J_C_) at which it is achieved and the collector-base bias (V_CB_). GaAs substrates were having an intentional defect density as specified in the table.

Ref.	Substrate	TDD	A_E_	Emitter	Base Doping & Thickness		β_max_	J_C_	V_CB_
		(cm^−2^)	(µm^2^)		(cm^−3^)	(nm)		(mA/µm^2^)	(V)
Won [32]	Si	–	2500	AlGaAs	1 × 10^19^	150	45	0.02	(0)
Ito [30]	GaAs *	≤3 × 10^5^ 10^8^	13	AlGaAs	1 × 10^19^	^†^ 80	90 25	0.25	(0)
Liu [31]	Si	–	80–160 40	AlGaAs	5 × 10^18^	100 ^†^ 100	45 ~100	0.1 1	0
Heidelberger [29]	(Ge/)Si	~2 × 10^7^	2827	InGaP	7 × 10^17^	90	60	0.035	0
Loke [33]	(Ge/)Si	~2 × 10^7^	2000	InGaP	1.9 × 10^19^	55	95	0.05	3.3
Loke [34]	(Ge/)Si	~2 × 10^7^	48	InGaP	3 × 10^19^	55	55	0.08	0.3
**NRref**	Si	<10^6^	531	InGaP	7.5 × 10^19^	20	31	5.7 × 10^−4^	0
**NRbase**	Si	<10^6^	208	InGaP	3 × 10^19^	20	112	1.2 × 10^−3^	0

* The GaAs substrates used had an intentional defect density indicated by the number in the TDD column. **^†^** This device has a compositionally graded AlGaAs base.

## Data Availability

Not applicable.
